# Short-Term Exposure of *Mytilus coruscus* to Decreased pH and Salinity Change Impacts Immune Parameters of Their Haemocytes

**DOI:** 10.3389/fphys.2018.00166

**Published:** 2018-03-06

**Authors:** Fangli Wu, Zhe Xie, Yawen Lan, Sam Dupont, Meng Sun, Shuaikang Cui, Xizhi Huang, Wei Huang, Liping Liu, Menghong Hu, Weiqun Lu, Youji Wang

**Affiliations:** ^1^National Demonstration Center for Experimental Fisheries Science Education, Shanghai Ocean University, Shanghai, China; ^2^International Research Center for Marine Biosciences at Shanghai Ocean University, Ministry of Science and Technology, Shanghai, China; ^3^Key Laboratory of Exploration and Utilization of Aquatic Genetic Resources, Ministry of Education, Shanghai Ocean University, Shanghai, China; ^4^Department of Biological and Environmental Sciences, Sven Lovén Centre for Marine Infrastructure-Kristineberg, University of Gothenburg, Fiskebäckskil, Sweden; ^5^Key Laboratory of Marine Ecosystem and Biogeochemistry, Second Institute of Oceanography, State Oceanic Administration, Hangzhou, China; ^6^State Key Laboratory of Satellite Ocean Environment Dynamics, Second Institute of Oceanography, State Oceanic Administration, Hangzhou, China

**Keywords:** acidification, salinity, *Mytilus coruscus*, flow cytometry, immune response, haemocyte

## Abstract

With the release of large amounts of CO_2_, ocean acidification is intensifying and affecting aquatic organisms. In addition, salinity also plays an important role for marine organisms and fluctuates greatly in estuarine and coastal ecosystem, where ocean acidification frequently occurs. In present study, flow cytometry was used to investigate immune parameters of haemocytes in the thick shell mussel *Mytilus coruscus* exposed to different salinities (15, 25, and 35‰) and two pH levels (7.3 and 8.1). A 7-day *in vivo* and a 5-h *in vitro* experiments were performed. In both experiments, low pH had significant effects on all tested immune parameters. When exposed to decreased pH, total haemocyte count (THC), phagocytosis (Pha), esterase (Est), and lysosomal content (Lyso) were significantly decreased, whereas haemocyte mortality (HM) and reactive oxygen species (ROS) were increased. High salinity had no significant effects on the immune parameters of haemocytes as compared with low salinity. However, an interaction between pH and salinity was observed in both experiments for most tested haemocyte parameters. This study showed that high salinity, low salinity and low pH have negative and interactive effects on haemocytes of mussels. As a consequence, it can be expected that the combined effect of low pH and changed salinity will have more severe effects on mussel health than predicted by single exposure.

## Introduction

The burning of fossil fuels has greatly increased the CO_2_ concentration in the atmosphere. A significant part of this CO_2_ is absorbed by the ocean leading to seawater carbonate chemistry changes (Doney and Schimel, 2007; Webb et al., 2016). The process of the seawater surface pH decline and acidity increase is known as ocean acidification (OA) (Brierley and Kingsford, 2009). It is projected that by the end of this century, the average surface seawater pH will decrease by 0.3–0.4 (Fabry et al., 2008). Many studies have shown that OA adversely affects various biological processes in bivalves including fertilization (e.g., Shi et al., 2017a,b), behavior (e.g., Peng et al., 2017), and molecular responses (e.g., Zhao et al., 2017). OA also influences immune function (Wang et al., 2016), calcification (Zhang et al., 2007), and metabolism (Clark et al., 2013) in many other shellfish species. In addition, short-term pH decrease has been reported to affect defense responses (Sui et al., 2017b), energy budget (Sui et al., 2016), and antioxidant response (Sui et al., 2017a) of mussels. All together, these data suggest that OA will affect the healthy physiological activities of marine species (Lewis et al., 2016).

Salinity is a key environmental factor affecting growth and health of marine mussels. For example, freshwater imports and heavy rains in coastal waters can increase mussel death (Cheung, 1991; Gajbhiye and Khandeparker, 2017). Many studies have shown that changes in salinity influence the respiration (e.g., Stickle and Sabourin, 1979), hormone secretion (e.g., Lacoste et al., 2001), heart rate (e.g., Bakhmet et al., 2005; Braby and Somero, 2006), growth rate (e.g., Westerbom et al., 2002), and energy acquisition (e.g., Gardner and Thompson, 2001) in bivalves. Low salinity significantly reduces the total haemocyte count and phagocytosis in *Mytilus edulis* (Bussell et al., 2008). Salinity also impacted the total haemocyte count, cell volume, phagocytosis, lysozyme-like activity and superoxide dismutase activity of the clam *Chamelea gallina* (Matozzo et al., 2007; Monari et al., 2007). Haemocyte mortality, esterase and phagocytosis in the Pacific oyster *Crassostrea gigas* were negatively impacted by changes in salinity (Gagnaire et al., 2006a).

The thick shell mussel *Mytilus coruscus* is an important ecological and economic shellfish species in China, mainly distributed in the coastal waters of East China Sea and Yellow Sea (Qin et al., 2014; Liu et al., 2015). The Zhoushan Islands sea area is one of the largest aquaculture areas of the thick shell mussel in China (Liao et al., 2013), with a total area of 1,300 hm^2^ and an annual production of 500,000 tons for a value of 500 million RMB (Cheng, 2014). Around the Zhoushan Islands sea area, mussels often experiences short-term fluctuations of salinity (15–35‰) and pH (7.3–8.1) leading to increased mortality during the wet season. These conditions are expected to increase in frequency and intensity due to global climate changes and OA. As a consequence, it is important to better understand the impact of salinity and pH on *M. coruscus*.

Immune response is a key physiological parameter with consequences for bivalve fitness and survival in the face of environmental changes. Cellular immunity is one of the most important lines of defense in shellfish, and the response ability to environmental changes is largely dependent on the haemocyte function (Mydlarz et al., 2006). Haemocytes play crucial roles in mollusk immune defense, including killing pathogens and swallowing foreign substances (Donaghy et al., 2009; Kwon et al., 2014). Previous studies have demonstrated that many biotic and abiotic factors can alter haemocyte-dependent defense mechanisms in bivalves, thus reducing their immune defense capacities (Matozzo et al., 2007). Bivalves are known to be sensitive to environmental factors such as pH (Berge et al., 2006; Bibby et al., 2008; Cole et al., 2016), dissolved oxygen (Sui et al., 2016), temperature and salinity (Gagnaire et al., 2006a; Cole et al., 2016). However, these factors are often studied in isolation neglecting their potential interactions. For example, only a few studies have tested how exposure to decreased pH impacts sensitivity of mussels to other stressors such as high temperature, oxygen and metal contaminants (but see Lewis et al., 2013; Huang et al., 2016; Sui et al., 2016).

Most studies on bivalve haemocyte immunity were performed using a single experimental method and focusing on single environmental factor. The aim of this article was to use a full factorial design to evaluate the impact of salinity and pH, alone or in combination. *M. coruscus* were exposed to varying salinity (15, 25, and 35‰) and pH (pH 8.1, and pH 7.3) for 7 days (*in vivo*) or 5-h (*in vitro*). *In vivo* experiments refer to the use the live animals while *in vitro* experiments refers to the use *ex vivo* cells, tissues, or organs (Johnston et al., 2010; Magdolenova et al., 2014). *In vitro* experiments were carried out to minimize the cushioning or shielding effects provided by the organism during osmotic adjustment (Shumway, 1977; Harris and Aladin, 1997), thus obtaining the immediate haemocyte responses to environmental changes. The *in vivo* approach was also used to understand the haemocyte response when they are housed within the body. Total haemocyte counts (THC), haemocyte mortality (HM), phagocytosis (Pha), esterase activity (Est), reactive oxygen species production (ROS), and lysosomal content (Lyso) were measured using flow cytometry.

## Materials and methods

### Experimental animals

Thick shell mussels (shell length, 55 ± 5 mm) were collected from a mussel raft at the Shengsi island of Zhejiang Province (121° 49′59.757″ E, 30° 33′00.945″ N), China in August 2016 (seawater temperature 23–25.0°C; salinity 25.0‰; pH 8.11). Mussels with no shell damage were selected and epibionts on the shell were softly removed. All collected mussels were cultured in open-flow tanks (500 l) equipped with air supply and filtration systems, and seawater conditions were mimicking the sampling site at sampling: temperature 25.0°C, salinity 25.0‰ and pH 8.1. The mussels were fed with the microalgae algae *Chlorella spp*. (concentration: 2.5 × 10^5^ cells ml^−1^) twice daily. Prior to the experiment, the mussels were allowed to acclimate to laboratory conditions for 2 weeks. The handling of experimental animals was carried on in terms of regulations of the animal welfare for scientific research made by the Institutional Animal Care and Use Committee (IACUC) of Shanghai Ocean University.

### Experimental design

A full factorial design was used to test the combined effects of pH and salinity. Large pH (pH ranging from 8.1 to 7.3) and salinity fluctuations (15–35) are observed at our sampling area, the Shengsi Island in summer time. Our treatments were selected to cover this natural variability: (i) two pH (8.1 vs. 7.3) and, (ii) three salinities (15, 25, and 35‰) for a total of six treatments.

### *In vivo* experiment

For the *in vivo* experiment, mussels were randomly divided into six treatments with three replicates (*n* = 30 mussels) per treatment. A flow-through system (240 l h^−1^) was used to minimize any interference from the metabolic waste products of the mussels, and tanks were covered with acrylic plates to reduce external disturbance. Mussels were fed with *Chlorella spp*. as described above during the experiment. Mussels were slowly transferred to new experimental conditions by a gradual decrease in pH values from 8.1 to 7.3 and a gradual decrease/increase in seawater salinities from 25 to 15‰/35‰ over 5 days. Then, haemocyte parameters were measured after a seven days exposure. pH was measured and controlled by addition of pure CO_2_ using a pCO_2_/pH system (DAQ-M, 4 Channel, Loligo® Systems Inc., Tjele, Denmark) equipped with WTW pH 3310 meters and SenTix 41 pH electrodes (Loligo Systems Inc., Tjele, Denmark) and operated by CapCTRL software (Loligo Systems Inc., Tjele, Denmark). pH was also measured daily using a portable pH meter (pH-201, MSITECH (Asia-Pacific) Pte. Ltd., Singapore) calibrated with the NBS scale. Different salinities (15, 25, and 35‰) were achieved by diluting seawater (35‰, autoclaved and filtered) with Milli-Q water and measured using ATAGO refractometer. Salinity was measured by a multipara meter instrument (model 5200A, YSI, USA). Total alkalinity (TA) was determined by titration. Other parameters of the seawater carbonate chemistry [pCO_2_, dissolved inorganic carbon (DIC), calcite saturation state (Ωcal) and aragonite saturation state (Ωara)] were calculated from TA and pH_NBS_ using CO_2_SYS (Lewis et al., 2013).

Haemolymph was collected after 1, 2, 4, and 7 days from the posterior adductor muscle of mussel using a 3.0 ml plastic syringe with a 22 G needle and all samples were stored on ice to minimize aggregation of haemocytes. For reducing individual variation, haemolymph from six mussels (1.0–1.5 ml each mussel) sampled in the same aquarium were pooled. For each pool, A Multisizer™ 3 Coulter Counter electronic particle counter/size analyzer (Beckman Coulter) was used to determine the haemocyte concentration (amount of cells per ml) (Wang et al., 2014). To measure THC, 9.5 ml Isoton®II solution and 0.5 ml haemolymph were mixed together, and 1,000 μl of the mixed solution was counted each time and repeated four times. Data were analyzed by Multisizer™ 3 software (Beckman Coulter, Inc., • 4300 N.Harbor Boulevard, Box 3100 • Fullerton, California 92834–3100, USA).

### *In vitro* experiment

An *in vitro* experiment was carried following the same experimental design for aduration of 5 h. Haemocytes from 30 mussels were pooled and prepared following Gagnaire et al. (2006a) without the excetion of the centrifugation step to limit the mortality of haemocytes. Haemolymph was diluted (1:9 in filtered autoclaved sea water) with water corresponding to the six treatments and prepared as described in section Experimental Design. Samples were collected after 2 and 5 h.

### Flow cytometry

#### Flow cytometry setup

A BD Accuri™ C6 flow cytometer (BD Biosciences, USA) with an air cooled argon laser was used to analyze the haemocyte parameters. A forward scatter (FSC) threshold was set in order to eliminate cell debris and bacteria. Data were visualized as cell cryptograms showing the granularity (SSC value), the relative size (FSC value), and the fluorescence channels corresponding to the fluorescent markers used. Each sample analysis included a total of 20,000 events, and the speed was maintained as a total event less than 300 s^−1^.

#### Parameter measurements

The type of fluorescence recorded depended on the parameter monitored: ROS, Pha, Lyso and Est were measured at FL1, and HM was tested at FL2 (Wang et al., 2012a). Haemocyte mortality (HM) was tested using propidium iodide (PI, 1.0 mg ml^−1^, Sigma Aldrich) and was evaluated as the percentage of haemocytes showing PI fluorescence relative to total haemocyte counts. Briefly, 400 μl haemolymph and 10 μl of of PI were mixed together in the dark for 30 min before a flow cytometer analysis at 4°C for a final concentration of 50 μg ml^−1^. Phagocytosis (Pha) was measured by the percentage of cells that had engulfed at least three fluorescent beads relative to all cells (Gagnaire et al., 2006b). Briefly, 400 μl haemolymph were incubated with 10 μl of a 1/10 dilution of Fluorospheres® carboxylate-modified microspheres (diameter 1.0 μm, yellow–green fluorescent, Invitrogen) for 1 h in the dark at ambient temperature. Non-specific esterase (Est) activity was evaluated using fluorescein diacetate (FDA, Sigma), which was evaluated as the percentage of fluorescent cells relative to all cells (Gagnaire et al., 2008). An aliquot of the 400 μl haemolymph was incubated with 2 μl of the 1/10 dilution of FDA (400 μM), and then the haemolymph samples were incubated at ambient temperature for 15 min in the dark before measurements. ROS was assessed by 2′7′-dichlorofluorescein diacetate (DCFH-DA; Sigma), which was defined on the basis of fluorescent cells among all cells (Delaporte et al., 2003) and expressed in arbitrary units (AU). Each analysis, 4 μl of DCFH-DA was added to the 400 μl of the haemolymph samples incubated for 15 min in the dark at room temperature for ROS detection. Lysosomal content (Lyso) was measured by a yellow fluorescent dye (LysoTracker® Yellow HCK-123, 1 mM in DMSO, Invitrogen), which was expressed in mean intensity of LysoTracker fluorescence exhibited by all the haemocytes in arbitrary units (AU) (Gagnaire et al., 2008). One μl of a LysoTracker was added to 400 μl haemolymph, and the suspension was incubated for 2 h at room temperature in the dark.

### Statistical analyses

The SPSS 17.0 software package was used for all statistical analyses. Data were checked for normality using the Shapiro–Wilk's test and for homogeneity of variance using the Levene's test. The effects of pH, salinity, time and their interactions were analyzed using a three-way ANOVAs. When an interaction was observed between the tested parameters, an one-way ANOVA was carried out for evaluating time effects at fixed salinity and pH level *in vivo* experiment, the salinity effects at fixed time and pH level *in vivo* and *vitro* experiment followed by Tukey's HSD post-hoc multiple range tests. For the same reason, Student's *t*-test was applied to test the difference between the two pH levels at each time point and fixed salinity level and the difference between two time points at each pH and fixed salinity level. Finally, a principal component analysis (PCA) was carried out for each measured parameter using XLSTAT®2014. A biplot was built with both the measured parameters and the observations. For all analysis, the results are expressed as the means ± SD and significant differences were set at *p* < 0.05.

## Results

### Seawater chemistry parameters

Seawater chemistry for each treatment is summarized in Table 1. Over the 7 days of the *in vitro* experiment, seawater temperature was maintained at 25.0 ± 0.7°C, pH and salinity were stable within each treatments, and TA ranged from 2,209 to 2,373 μmol kg^−1^.

**Table 1:** Seawater carbonate chemistry (mean ± SD) for the experiment.

**Treatments**	**Salinity**	**Temperature**	**pHNBS**	**TA**	**DIC**	**pCO_2_**	**Ωcal**	**Ωara**
pH*Salinity	**(psu)**	**(°C)**		**(μmol kg^−1^)**	**(μmol kg^−1^)**	**(μatm)**		
8.1 * 15	15.1 ± 0.1	25.1 ± 0.1	8.10 ± 0.02	2,220 ± 13	2,047 ± 16	431 ± 20	4.23 ± 0.15	2.54 ± 0.09
7.3 * 15	15.0 ± 0.1	24.9 ± 0.1	7.31 ± 0.02	2,314 ± 11	2,379 ± 11	3,195 ± 129	0.81 ± 0.03	0.49 ± 0.02
8.1 * 25	25.1 ± 0.2	25.0 ± 0.1	8.10 ± 0.02	2,253 ± 17	1,998 ± 12	365 ± 19	5.06 ± 0.23	3.23 ± 0.14
7.3 * 25	25.1 ± 0.1	25.0 ± 0.2	7.30 ± 0.02	2,348 ± 18	2,382 ± 20	2,899 ± 134	1.01 ± 0.03	0.65 ± 0.02
8.1 * 35	35.1 ± 0.2	25.0 ± 0.2	8.11 ± 0.02	2,254 ± 14	1,915 ± 16	319 ± 18	5.68 ± 0.19	3.75 ± 0.12
7.3 * 35	34.9 ± 0.2	25.1 ± 0.2	7.30 ± 0.01	2,350 ± 11	2,355 ± 17	2,722 ± 108	1.19 ± 0.04	0.78 ± 0.03

*Temperature (°C), salinity (psu), and total alkalinity (TA; μmol kg^−1^) were measured on each sampling date (n = 4). pH_NBS_ was measured continuously during the experiment. Dissolved inorganic carbon (DIC), partial pressure of CO_2_ in seawater (pCO_2_) and saturation state for calcite (Ωcal) and aragonite (Ωara) were calculated from the measured temperature, salinity, pH and TA*.

### *In vitro* experiments

**Figure 1:**
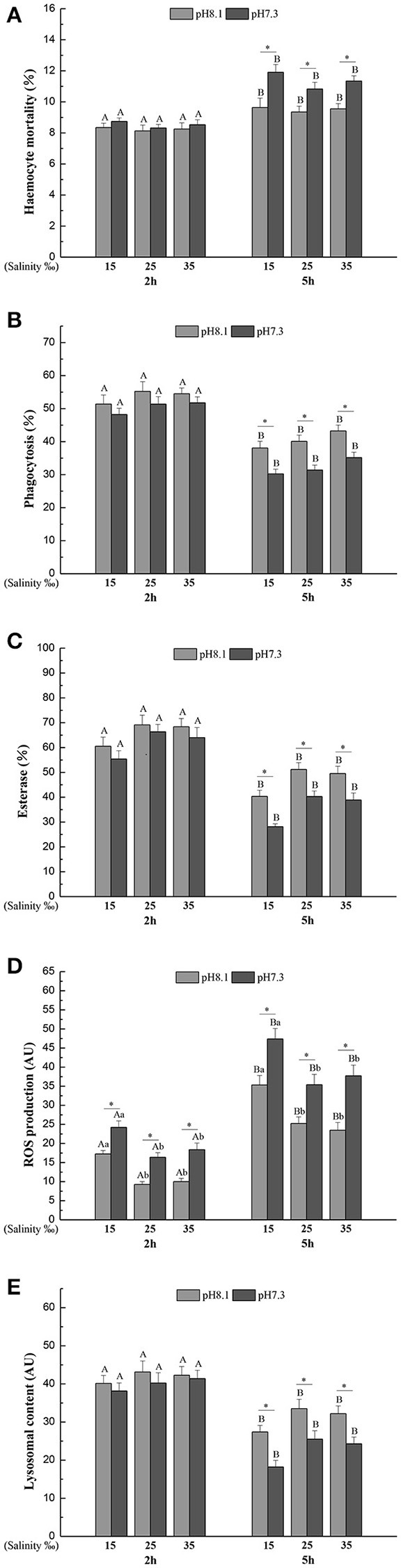
**(A)** Haemocyte mortality (HM), **(B)** Phagocytosis (Pha), **(C)** Esterase (Est), **(D)** Reactive oxygen species (ROS), **(E)** Lysosomal content (Lyso) of *M. coruscus* exposed to six combinations of salinity (15, 25, and 35‰) and pH (7.3 and 8.1) at 2 and 5 h for *in vitro* experiment. Different capital letters indicate significant differences among time points within each salinity level in pH 7.3 level or control group (*p* < 0.05). Different small letters indicate significant differences between salinity within each time point in pH 7.3 level or control group (*p* < 0.05). Asterisk indicates significant differences between pH within each time point and fixed salinity treatment (*p* < 0.05).

Haemocyte mortality (HM), phagocytosis (Pha), Esterase (Est), and lysosomal content (Lyso) were significantly affected by time, pH, salinity, and interaction of pH and time (Table 2). HM significantly increased and Pha, Est, and Lyso significantly decreased when haemocytes were exposed to each pH and salinity for 5 h. Low pH induced significantly higher HM and lower Pha, Est, and Lyso at each salinity level after 5 h (Figures 1A–C,E).

**Table 2:** Three-way ANOVA summary on effects of pH, salinity (S) and time (T) on haemocyte mortality (HM), phagocytosis (Pha), esterase (Est), reactive oxygen species (ROS), and lysosomal content (Lyso) of *M. coruscus in vitro* experiments.

**Source**	**df**	**HM**			**Pha**			**Est**			**Ros**			**Lyso**		
		***MS***	***F***	***P***	***MS***	***F***	***P***	***MS***	***F***	***P***	***MS***	***F***	***P***	***MS***	***F***	***P***
S	2	0.748	5.157	0.014	53.523	13.348	<0.001	396.715	42.575	<0.001	331.057	87.955	<0.001	76.355	15.696	<0.001
pH	1	10.209	70.381	<0.001	295.470	73.689	<0.001	529.815	56.860	<0.001	867.636	230.513	<0.001	238.416	49.011	<0.001
T	1	37.872	261.092	<0.001	2219.445	553.517	<0.001	4582.141	491.754	<0.001	2972.34	789.682	<0.001	1772.762	364.430	<0.001
S * pH	2	0.188	1.297	0.292	0.700	0.175	0.841	2.670	0.287	0.753	5.558	1.477	0.248	1.300	0.267	0.768
S * T	2	0.099	0.681	0.516	9.863	2.460	0.107	2.955	0.317	0.731	14.375	3.819	0.036	13.411	2.757	0.084
pH * T	1	5.460	37.643	<0.001	54.932	13.700	0.001	115.286	12.372	0.002	49.024	13.025	0.001	93.679	19.258	<0.001
S * pH * T	2	0.067	0.464	0.634	0.095	0.024	0.977	0.612	0.066	0.937	1.600	0.425	0.658	0.985	0.203	0.818

*pH: 7.3 and 8.1; salinity: 15, 25, and 35‰; T: 2 h, 5 h. P = p-value*.

Reactive oxygen species (ROS) was significantly affected by time, pH and salinity, and there were two significant interactions of salinity and time, pH and time (Table 2). After 5 h, ROS significantly increased when the haemocytes were exposed to each pH and salinity level. Low pH treatment led to a significant increase in ROS at each time point and salinity level. Low salinity treatment significantly increased ROS at each time point and pH level (Figure 1D).

### *In vivo* experiments

**Figure 2:**
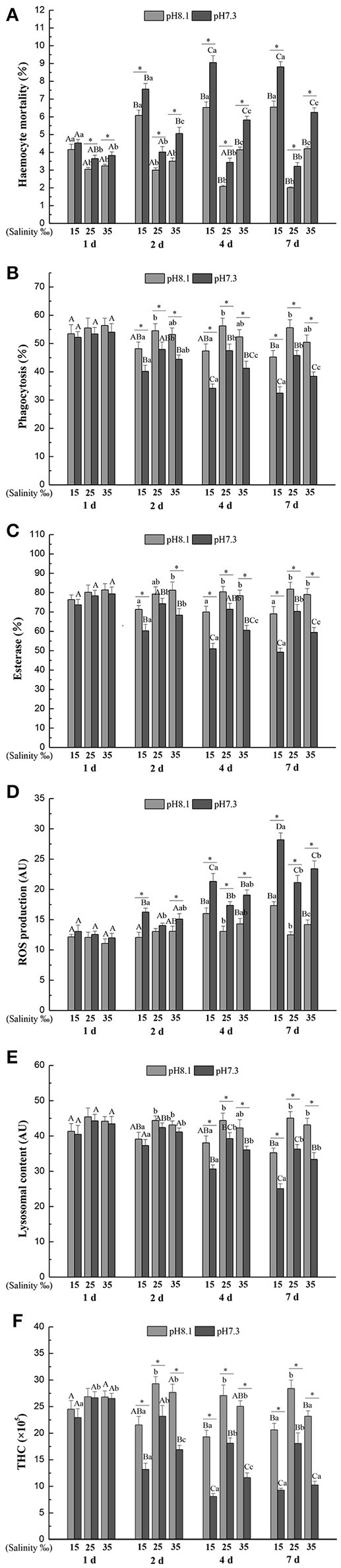
**(A)** Haemocyte mortality (HM), **(B)** Phagocytosis (Pha), **(C)** Esterase (Est), **(D)** Reactive oxygen species (ROS), **(E)** Lysosomal content (Lyso), **(F)** Total haemocyte count (THC) of *M. coruscus* exposed to six combinations of salinity (15, 25, and 35‰) and pH (7.3 and 8.1) at 1, 2, 4, and 7 days for *in vivo* experiment. Different capital letters indicate significant differences among time points within each salinity level in pH 7.3 level or control group (*p* < 0.05). Different small letters indicate significant differences between salinity within each time point in pH 7.3 level or control group (*p* < 0.05). Asterisk indicates significant differences between pH within each time point and fixed salinity treatment (*p* < 0.05).

Haemocyte mortality (HM) was significantly affected by time, pH and salinity and all their interactions (Table 3). Overall, HM was higher when the mussels were exposed in low or high salinities for each time point and pH level. High salinity led to higher HM than low salinity. Low pH treatment led to a significant increase in HM at each time point and salinity level except for low salinity at day 1 (Figure 2A).

**Table 3:** Three-way ANOVA summary on effects of pH and salinity, salinity (S) and time (T) on haemocyte mortality (HM), phagocytosis (Pha), esterase (Est), reactive oxygen species (ROS), lysosomal content (Lyso). and total haemocyte counts (THC) of *M. coruscus in vivo* experiments.

**Source**	**df**	**HM**			**Pha**			**Est**			**Ros**			**Lyso**			**THC**		
		***MS***	***F***	***P***	***MS***	***F***	***P***	***MS***	***F***	***P***	***MS***	***F***	***P***	***MS***	***F***	***P***	***MS***	***F***	***P***
S	2	78.674	1367.490	<0.001	378.005	64.025	<0.001	894.881	93.062	<0.001	41.784	57.794	<0.001	296.039	96.220	<0.001	316.969	178.975	<0.001
pH	1	34.825	605.327	<0.001	1171.520	198.426	<0.001	2182.901	227.009	<0.001	346.241	478.915	<0.001	392.467	127.562	<0.001	1139.836	643.603	<0.001
T	3	8.374	145.550	<0.001	305.660	51.771	<0.001	391.272	40.690	<0.001	186.752	258.312	<0.001	163.250	53.060	<0.001	229.364	129.509	<0.001
S * pH	2	0.600	10.425	<0.001	7.021	1.189	0.313	79.306	8.247	0.001	4.521	6.253	0.004	0.933	0.303	0.740	13.019	7.351	0.002
S * T	6	5.847	101.638	<0.001	34.096	5.775	<0.001	51.013	5.305	<0.001	11.253	15.564	<0.001	13.980	4.544	0.001	17.347	9.795	<0.001
pH * T	3	1.767	30.719	<0.001	87.800	14.871	<0.001	199.841	20.782	<0.001	66.287	91.686	<0.001	72.316	23.504	<0.001	14.787	64.814	<0.001
S * pH * T	6	0.193	3.352	0.008	2.092	0.354	0.904	8.631	0.898	0.504	0.659	0.911	0.495	0.597	0.194	0.977	1.974	1.114	0.368

*pH: 7.3 and 8.1; salinity: 15, 25, and 35‰; T: 1, 2, 4, and 7 days. P = p-value*.

Phagocytosis (Pha) was significantly affected by time, pH and salinity, and the interaction of salinity and time, and the interaction between pH and time (Table 3). Low pH treatment led to a significant decrease in Pha at each time point and salinity level except for day 1. Pha tend to decrease with time at each salinity and pH level except for pH 8.1 and salinity 25. Low salinity led to a significant decrease in Pha at each time and pH treatment except for day 1, while high salinity only significantly reduced Pha under low pH level at day 4 and 7 (Figure 2B).

Salinity, time, pH and their interactions had significant effects on esterase (Est) throughout the experiment (Table 3). Est significantly decreased with time under low pH level at each salinity treatment. Low pH significantly decreased Est at each time and salinity treatment except for day 1 and pH 8.1 and salinity 25 at day 2. Low salinity resulted in a significant reduction in Est at each time and pH treatment except day 2 at pH 8.1 and day 1, whereas high salinity only significantly decreased Est under low pH condition at day 4 and day 7 (Figure 2C).

Salinity, time, pH and their interactions had significant effects on reactive oxygen species (ROS) over the experimental period (Table 3). In general, ROS significantly increased with time at each salinity and pH level except the control. ROS was significantly higher when the mussels were exposed to low pH at each time and salinity treatment except day 1 and pH 8.1 and salinity 25 at day 2. Low salinity significantly increased ROS under pH 7.3 level at day 2, 4, and 7 and under pH 8.1 level at day 4 and 7, whereas high salinity only significantly increased ROS under low pH condition at day 7 (Figure 2D).

The lysosomal content (Lyso) was significantly influenced by time, pH and salinity, with an interaction between salinity and time and an interaction between pH and time (Table 3). Lyso generally significantly decreased with time at each salinity and pH level except for pH 8.1/salinity 25. From day 4, low pH led to a significant decrease in Lyso at each salinity treatment. Low salinity treatment significantly decreased Lyso at each time and pH treatment except day 1, whereas high salinity has no significant effect on Lyso at each time and pH treatment (Figure 2E).

Total haemocyte count (THC) was significantly affected by time, pH and salinity and their interactions (Table 3). THC generally significantly decreased with time at each salinity and pH level except for pH 8.1/salinity 25. From day 2, low pH treatment resulted in a significant decrease in THC at each salinity treatment. Low salinity treatment significantly decreased THC at each time and pH treatment except the pH 8.1 level at 1 d, whereas high salinity only significantly decreased THC under low pH condition at day 2, 4, and 7 and under pH 8.1 at day 7 (Figure 2F).

### Principal component analysis

**Figure 3:**
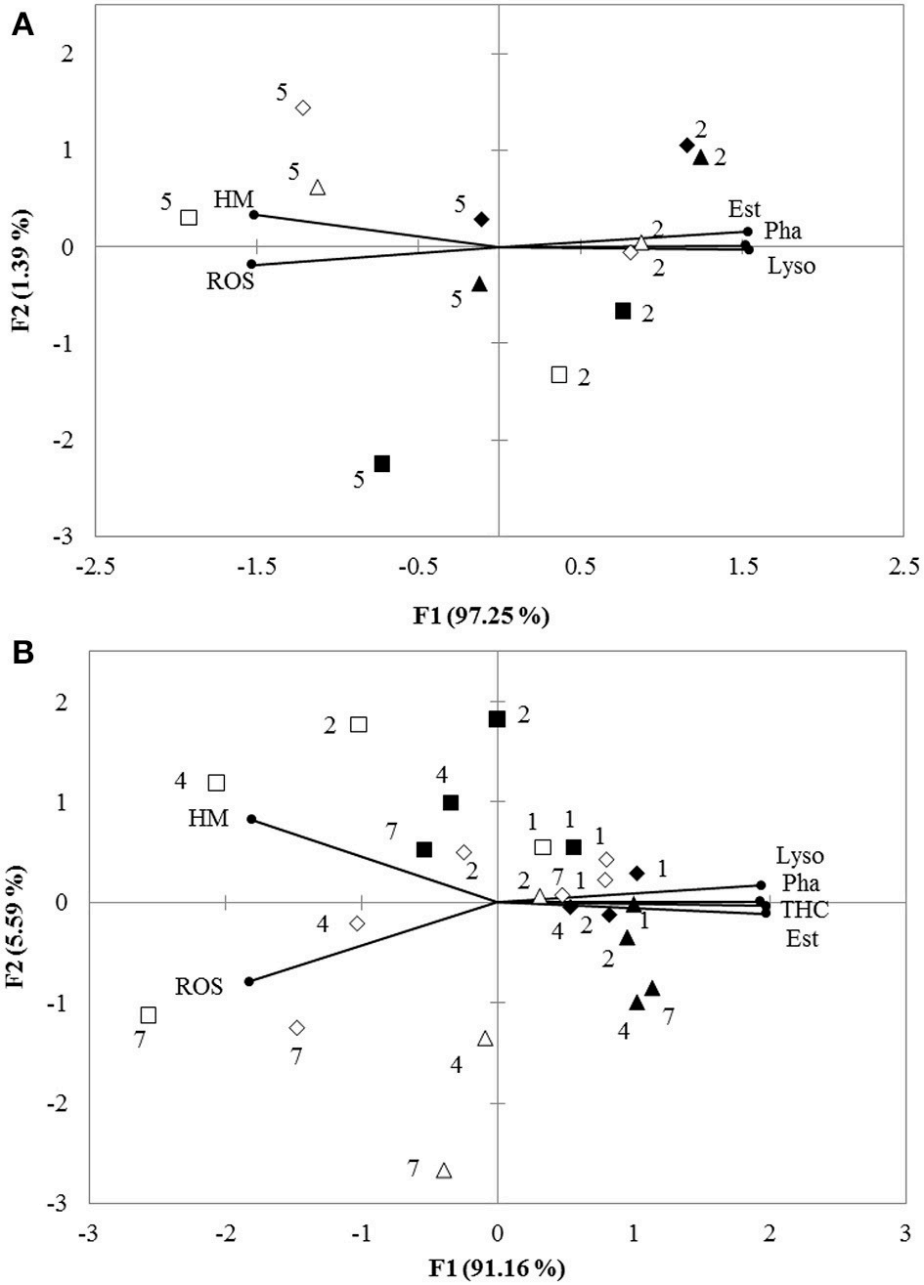
**(A)** Biplot originating from PCA integrating all measured variables (HM, Pha, Est, ROS, and Lyso) and two time points (hours: 2 and 5) at six different treatments (■−15‰ × pH 8.1, □−15‰ × pH 7.3, ▴−25‰ × pH 8.1, Δ-25‰ × pH 7.3, ♦−35‰ × pH 8.1, ♢−35‰ × pH 7.3) for *in vitro* experiment. **(B)** Biplot originating from principal component analysis integrating all measured variables (HM, Pha, Est, ROS, Lyso, and THC) and four time points (days: 1, 2, 4, and 7) at six different treatments (■−15‰ × pH 8.1, □−15‰ × pH 7.3, ▴−25‰ × pH 8.1, Δ-25‰ × pH 7.3, ♦−35‰ × pH 8.1, ♢−35‰ × pH 7.3) for *in vivo* experiment. Both the loadings of the variables (•) and the scores of the experimental conditions were shown.

Principal component analysis (PCA) revealed that 98.64 and 96.75% of overall variance were explicated by the two principal components for the *in vitro* and *in vivo* experiments, respectively. For the *vitro* experiment, PC1 explained 97.25% of overall variance, showing the most significant result referred to the separation between low pH and normal pH treatment. This axis presented a pH specific response since the low pH 7.3 was separated from the normal pH. THC, Pha, Lyso, and Est were grouped together and negatively correlated with HM and ROS (Figure 3A). For the *vivo* experimental period, PC1 expressed 91.16% of the overall variance, showing a similar trend to the *in vitro* experiment (Figure 3B).

## Discussion

Our results show that a short-term exposure to changes in salinity and seawater pH has an effect on haemocyte parameters related to immune functions in mussels. *In vitro* experiments are designed to investigate the immediate response of haemocytes to ocean acidification and varying salinity in this study. These effects could be explained by a decreased haemocyte activity as a result of osmotic effects (Pipe and Coles, 1995) or a physiological inhibition due to decreased respiration, and increased activity of antioxidant enzymes (Matozzo et al., 2013). Similar trends were observed in both *in vivo* and *in vitro* experiments. However, haemocyte immune parameters (HM, Pha, ROS, and Lyso) were higher in the *in vitro* experiments as compared to the *in vivo* experiments. This can be explained by the lack of buffering by the organisms and direct exposure of the haemocytes to environmental changes in the *in vitro* experiment.

HM values were low in all treatment groups over the whole experiment period. This is consistent with other studies on Pacific oyster *Crassostrea gigas* (Delaporte et al., 2003) and Eastern oyster *Crassostrea virginica* (Ashton-Alcox and Ford, 1998). Assessing the mortality of haemocytes with propidium iodide relies on the integrity of the cytomembrane and it is then difficult to differentiate between apoptotic and necrotic cells. We observed that HM was significantly increased when mussels were exposed to decreased pH, indicating a high sensitivity of haemocytes to pH. A similar effect was observed in the oyster *C. gigas* (Wang et al., 2016). This increase in HM may be a consequence of an insufficient stress-induced antioxidant enzymes production, which enhanced formation of intracellular ROS and then resulted in apoptosis (Turrens, 2003). In comparison with pH, salinity changes had a lower impact on HM. An increase of HM exposed to salinity changes has also been found in the oyster *C. gigas* (Gagnaire et al., 2006a) and the short neck clam *Paphia malabarica* (Gajbhiye and Khandeparker, 2017). A significant interaction between pH and salinity was found leading to a stronger negative effect of the combined salinity and pH changes than what would be expected from the single stressors. This highlights the importance to study drivers in realistic combinations.

Phagocytosis is a key element of the cellular defense against foreign matter and pathogens. For example, mussels can effectively clear the debris from dead cells using phagocytosis (Hegaret et al., 2003). Environmental factors can affect phagocytosis in molluscs (Malagoli et al., 2008). In the present study, a reduction of phagocytosis under low pH and salinity changes was observed, suggesting negative impacts on the immunological competence of *M. coruscus*. Wang et al. (2016) found that seawater acidification led to reduced phagocytosis in the Pacific oyster *C. gigas*. Such a reduction of phagocytosis has also been found in the mussel *M. edulis* (Bibby et al., 2008). In comparison, salinity stress had a weaker effect on phagocytosis as compared with decreased pH. This suggests a higher tolerance of these mussels to salinity changes (Nel et al., 2015). A reduction of phagocytosis under low salinity condition was found in the mussel *P. viridis* (Wang et al., 2012b), the mussel *M. edulis* (Bussell et al., 2008), the oyster *C. gigas* (Gagnaire et al., 2006a), the abalone *H. diversicolor* (Cheng et al., 2004b) and the saltwater clam *C. gallina* (Matozzo et al., 2007). However, the intensity of the impact of salinity changes on phagocytosis may be species-specific (Wang et al., 2012a). Additionally, there was no significant interaction of salinity and pH suggesting that pH and salinity may have different mode of actions on haemocyte phagocytosis.

Our results show that low pH led to a decrease of esterase activity, a result consistent with our previous observations (Sui et al., 2016). Decreased pH had a stronger negative effect on esterase activity as compared with salinity changes. Esterase activity can be affected by pollutants and other environmental changes in salinity and pH (Pretti and Cognetti-Varriale, 2001; Wang et al., 2011; Xian et al., 2014). Gajbhiye and Khandeparker (2017) found a significant decrease in esterase activity when the short neck clam *Paphia malabarica* was exposed to salinity stress. Such a reduction of non-specific esterase activity has also been reported in the oyster *C. gigas* (Gagnaire et al., 2006a) and the mussel *P. viridis* (Wang et al., 2012a) at low salinity levels. Such changes in esterase activity are known to be the consequence of an oxidative stress (Paital and Chainy, 2010; Xian et al., 2014). Esterase is involved in the intracellular degradation in haemocytes (Mottin et al., 2010). Therefore, a decrease of esterase activity is likely caused by an increase of HM and ROS content.

Both decreased pH and salinity changes decreased ROS production inducing high oxidative defense in the mussel *M. coruscus*. ROS plays a key role in innate immune response through their microbicide role in bivalves (Terahara and Takahashi, 2008), leading to the generation of oxygen metabolites (Pipe, 1992). If ROS production is higher than antioxidant capacity, excessive ROS production may lead to oxidative damage to cells (Cheng et al., 2004a). As a consequence, the observed high ROS production may be a way to compensate for the reduction of haemocytes. A significant increase of ROS in response to exposure to decreased pH was observed in the oyster *C. gigas* (Wang et al., 2016) and the mussel *Mytilus coruscus* (Sui et al., 2016). In the present study, ROS production in haemocytes exposed to low and high salinity suggests that these stimulate the ability of haemocytes to produce ROS or inhibits some enzymes responsible for eliminating ROS (Lushchak, 2011). Contrasting results were found in the mussel *P. viridis* (Wang et al., 2012a), the abalone *Haliotis diversicolor supertexta* (Cheng et al., 2004b) and the clam *Paphia malabarica* (Gajbhiye and Khandeparker, 2017) where low salinities led to a reduction of ROS. Again, this may be a consequence of different sensitivities to changes in salinity between different species.

As the major bacteriolytic cellular organelle, lysosome hydrolyzes foreign bodies such as bacteria by releasing different hydrolytic enzymes (Monari et al., 2007) and is used for host defense as well as intracellular degradation (Olsen et al., 2003). We observed that Lyso was also significantly decreased in mussel haemocytes exposed to decreased pH and salinity changes. Wu et al. (2016) reported a similar results for low pH in the same species. In the mussel *Perna viridis* and in the clam *Paphia malabarica*, a significant decrease of lysosomal content was found under low salinity (Wang et al., 2012a; Gajbhiye and Khandeparker, 2017).

The immune system relies heavily on circulating haemocytes for immune surveillance in bivalves. The haemocyte concentrations are well correlated with external environmental factors such as salinity, pH and temperature (Wang et al., 2011, 2012a,b), and are considered as sensitive biomarker to monitor the effect of environmental disturbance. Our results have shown that decreased pH, salinity changes, and their interactions reduced THC. Matozzo et al. (2012) reported a strong negative impact of decreased pH on a THC and haemolymph lysozyme activity in bivalves. Sui et al. (2016) found that decreased pH also led to reduced THC in *M. coruscus*. Mussels exposed to salinity stresses showed reduced THC as a consequence of an osmotic pressure regulation or haemocyte lysis (Pipe and Coles, 1995). Similar studies showed that THC decreased under low salinities in the mussel *M. edulis* (Bussell et al., 2008), the mussel *P. viridis* (Wang et al., 2012a), the clam *Ruditapes philippinarum* (Reid et al., 2003), and the Taiwan abalone *Haliotis diversicolor supertexta* (Cheng et al., 2004b).

PCA identified pH as the main factor allowing to explain the difference between the measured parameters of the haemocytes. The two pH treatments were well separated by PC1 reflecting semblable immune responses in both *in vitro* and *in vivo* experiments. Comparison of ANOVA testing with PCA showed that the characteristics of haemocyte function associated with the decreased pH were lower Pha, Lyso, Est, and THC coupled with higher ROS and HM. This analysis did not allow to clearly separate the salinity treatments.

In conclusion, the present study combining two experimental approaches showed that *M. coruscus* exposed to changes in salinity and low pH is able to survive, although some haemocyte parameters are affected. Generally, both salinity changes, pH, and their interaction decrease have negative effects on haemocyte responses over time. Decreased pH had a stronger impact than salinity changes and the negative impact was enlarged when both stressors were combined. Overall, our results suggest that when exposed to the extremes of the present natural variability, mussels suffer sublethal negative effects that are likely to lead to reduced fitness. As a consequence of global changes, mussels will be exposed more frequently to these conditions as well as more several pH and salinity changes with potential lethal and sublethal consequences. At present, the physiological responses and mechanisms of marine mussels to multiple stressors have not been well clarified, and little is known about the interactions among the various environmental factors. Acidification and other factors that drive changes in marine ecosystems (e.g., temperature, dissolved oxygen, and salinity) may produce complex interactions, so it is necessary to explore the combined effects of multiple stressors from different levels. In addition, most studies investigated the short-term effects of ocean acidification on marine animals, and the ceo-physiological responses and adaptations of marine animals to long-term acidification are not clear. Future studies should consider long-term effects of ocean acidification in combination with other environmental changes on marine mussels as this can reflect their adaptations in coastal areas.

## Author contributions

Guarantor of integrity of entire study: FW and YW. Study concepts: FW, ZX, MH, SC, XH, and WH. Study design: FW, MH, and YW. Literature research: FW, ZX, YL, MS, SC, XH, and WH. Experimental studies: FW, ZX, YL, MS, SC, XH, and YW. Data acquisition and data analysis/interpretation: FW, ZX, and YW. Manuscript preparation: FW, LL, WL, and YW. Manuscript definition of intellectual content and manuscript editing: FW, SD, and YW. Manuscript revision/review: FW, SD, YW, and MH. Manuscript final version approval: FW, ZX, YL, SD, MS, SC, XH, WH, LL, MH, WL, and YW.

### Conflict of interest statement

The authors declare that the research was conducted in the absence of any commercial or financial relationships that could be construed as a potential conflict of interest.
